# Improved Insulin Sensitivity during Pioglitazone Treatment Is Associated with Changes in IGF-I and Cortisol Secretion in Type 2 Diabetes and Impaired Glucose Tolerance

**DOI:** 10.1155/2013/148497

**Published:** 2013-01-15

**Authors:** Lisa Arnetz, Neda Rajamand Ekberg, Charlotte Höybye, Kerstin Brismar, Michael Alvarsson

**Affiliations:** Department of Molecular Medicine and Surgery, D2:04, Karolinska Institutet and Karolinska University Hospital, 17176 Stockholm, Sweden

## Abstract

*Background*. Hypercortisolism and type 2 diabetes (T2D) share clinical characteristics. We examined pioglitazone's effects on the GH-IGF-I and HPA axes in men with varying glucose intolerance. *Methods*. 10 men with T2D and 10 with IGT received pioglitazone 30–45 mg for 12 weeks. OGTT with microdialysis in subcutaneous adipose tissue and 1 **μ**g ACTH-stimulation test were performed before and after. Glucose, insulin, IGF-I, IGFBP1, and interstitial measurements were analyzed during the OGTT. Insulin sensitivity was estimated using HOMA-IR. *Results*. HOMA-IR improved in both groups. IGF-I was initially lower in T2D subjects (*P* = 0.004) and increased during treatment (−1.4 ± 0.5 to −0.5 ± 0.4 SD; *P* = 0.007); no change was seen in IGT (0.4 ± 39 SD before and during treatment). Fasting glycerol decreased in T2D (*P* = 0.038), indicating reduced lipolysis. Fasting cortisol decreased in T2D (400 ± 30 to 312 ± 25 nmol/L; *P* = 0.041) but increased in IGT (402 ± 21 to 461 ± 35 nmol/L; *P* = 0.044). Peak cortisol was lower in T2D during treatment (599 ± 32 to 511 ± 43, versus 643 ± 0.3 to 713 ± 37 nmol/L in IGT; *P* = 0.007). *Conclusions*. Pioglitazone improved adipose tissue and liver insulin sensitivity in both groups. This may explain increased IGF-I in T2D. Pioglitazone affected cortisol levels in both groups but differently, suggesting different mechanisms for improving insulin sensitivity between T2D and IGT.

## 1. Introduction

Type 2 diabetes (T2D) is a significant public health issue due to its prevalence and complications. Obesity, particularly abdominal obesity, is a major risk factor for the disease. Central features of T2D include hyperglycemia, insulin resistance, and progressive *β*-cell failure. According to a theory developed by Björntorp et al., there are also disturbances in the central hormone axes, with activity increased in the hypothalamus-pituitary-adrenal (HPA) axis and decreased in the growth hormone (GH) insulin-like growth factor I (IGF-I) and LH-testosterone axes [1]. As T2D develops via an insidious phase of impaired glucose tolerance (IGT) [2], it is of importance to understand the pathogenesis of both conditions in order to improve treatment options and prevent progression of IGT to T2D. 

Of the end products produced by the central hormone axes, IGF-I and cortisol have the greatest effects on insulin sensitivity. IGF-I is produced mainly in the liver [3] and has effects highly comparable to those of insulin [4]. Its production is dependent on GH, insulin, and nutritional status [5]. Cortisol has anti-insulin effects on glucose metabolism, increasing gluconeogenesis and decreasing glucose uptake [6]. However, chronic elevation of glucocorticoid levels as well as hyperinsulinemia result in the accumulation of visceral fat [7]. Visceral adipose tissue is less sensitive to insulin action [8], leading to the increased lipolysis of triglycerides producing glycerol and free fatty acids (FFAs), which in turn inhibit GH secretion as do high glucose levels [5]. Increased levels of FFAs induce insulin resistance [9]. Cortisol interacts with the IGF system by binding to the corticoid-response elements on the IGF-binding protein 1 (IGFBP1) gene, stimulating transcription and thereby decreasing IGF-I bioavailability [10].

Altered insulin sensitivity in the adipose tissue appears to be an early and important disturbance in the development of IGT and T2D [11]. Insulin is a potent inhibitor of lipolysis, primarily through effects on hormone-sensitive lipase which hydrolyses triglycerides into FFAs and glycerol [12]. Hence, in insulin resistant states, FFAs are increased in serum and glycerol interstitially in subcutaneous (sc) adipose tissue, despite hyperinsulinemia [13]. Microdialysis is a technique for studying in vivo metabolism and can be used in sc adipose tissue to study glycolysis, via the analysis of glucose, pyruvate, and lactate, and lipolysis, via the analysis of glycerol [14]. 

Pioglitazone is a thiazolidinedione (TZD), a class of drugs used to treat T2D. Briefly, pioglitazone activates peroxisome-proliferator activated receptor gamma (PPAR*γ*) in a variety of cells, improving insulin sensitivity in the liver (decreasing gluconeogenesis) and in the adipose tissue as measured by the ability of insulin to inhibit lipolysis [15]. One of its key effects is the redistribution of fatty acids from visceral adipose tissue, skeletal muscles, and liver to the sc adipose tissue [15]. In a small clinical study, patients with hypercortisolism due to Cushing's disease responded well to a TZD [16]. This indicated that TZDs may constitute a useful tool for examining the effect of reduced insulin resistance on the HPA axis. Using microdialysis technique allows for the detailed study of insulin sensitivity in adipose tissue.

As cortisol is detrimental whereas IGF-I is beneficial to insulin sensitivity, the present study was designed to study the interaction between these hormones and glucose tolerance in overweight patients with IGT and T2D. The purpose of using pioglitazone treatment was not to evaluate its effect on insulin sensitivity per se, but to evaluate if it had any effect on the central hormone axes and how such effects may be related to insulin sensitivity of the liver and adipose tissue. As this was a pilot study, only males were studied to avoid the introduction of gender differences.

## 2. Materials and Methods

### 2.1. Subjects

Inclusion criteria were male gender and a BMI ≥ 28 kg m^−2^; exclusion criteria were a clinical history of congestive heart failure and for patients with T2D treatment with insulin or TZDs. 11 patients with T2D and 12 with IGT were enrolled in the study. One with T2D and two with IGT dropped out due to medication side effects; 10 in each group completed the study. The diagnosis of IGT was ascertained in the study's baseline oral glucose tolerance test (OGTT) according to the 1999 WHO criteria [17]; patients who did not fill the criteria for IGT were excluded without further testing.

### 2.2. Study Design

The study protocol was approved by the Regional Ethics Committee in Stockholm, Sweden, and the study was carried out in accordance with the Declaration of Helsinki. All subjects received information and gave written informed consent prior to entering into the study. The subjects were evaluated week zero using an OGTT and a low-dose adrenocorticotropic-hormone- (ACTH-) stimulation test (see below). They were then started on pioglitazone (Actos) 30 mg once daily in addition to their pre-existing medications. After four weeks, the daily pioglitazone dose was increased to 45 mg if no side effects were noted. Treatment continued for an additional eight weeks after which the two tests were repeated. The tests were performed on two separate days at all time points. All assessments were carried out at the research facility of the Department of Endocrinology, Metabolism and Diabetes at Karolinska University Hospital, initiated at 08:00 h after an overnight fast, and with the subject resting in the supine position. No tobacco or heavy exercise was permitted during the morning before either test.

### 2.3. Biometric Evaluations

Patients were examined by an MD at baseline and after 12 weeks of pioglitazone treatment. Their blood pressure, weight, and waist circumference were recorded at each evaluation.

### 2.4. Microdialysis and OGTT

Microdialysis technique has been described in detail previously [18]. A catheter was inserted into an antecubital vein and two microdialysis catheters, connected to microdialysis pumps, were inserted in paraumbilical sc adipose tissue. Low molecular weight substances in the interstitial fluid surrounding the catheters diffuse through a semipermeable membrane in the perfusion fluid, which is pumped through the catheter. The fluid (dialysate) is then collected in vials connected to the pump for analysis. Every 30 minutes, venous blood samples were drawn and the vials were changed. After a wash-in period of one hour, subjects were given 75 g glucose dissolved in 200 mL water to drink. Sample collection continued every 30 minutes for an additional 150 minutes. Due to lag time before interstitial fluid is transported through the microdialysis catheter to the collection vial, dialysate samples collected at any given time point were taken to reflect interstitial concentrations 30 minutes previously. Hence, fasting levels were analyzed from vials collected 30 minutes after glucose ingestion. Results from the catheter with the lowest lactate levels and least erroneous measurements were used in statistical analyses. 

The concentrations of interstitial glucose, lactate, pyruvate, and glycerol were measured using a CMA 600 analyzer (CMA microdialysis AB, Solna, Sweden); the methods of the analyses have been described in detail previously [19].

### 2.5. Low-Dose ACTH Test

The low-dose ACTH solution was prepared by removing 1 mL from a 50 mL bottle of NaCl 9 g/L and then adding to the 50 mL bottle 1 mL of 0.25 g/L solution synthetic ACTH (Synacthen; Novartis, Basel, Switzerland), resulting in a concentration of 250 *μ*g/50 mL = 0.005 g/L. A 0.001 g/L injection was prepared by drawing up 0.2 mL of the 0.005 g/L solution, and then 0.8 mL of pure NaCl solution. Blood samples were drawn from an iv catheter for the analysis of blood glucose and serum levels of cortisol, insulin, C-peptide, IGF-I, and IGFBP1 before the injection of the ACTH. Blood was drawn at 30, 60, and 90 minutes after the injection, for analyses of the same factors except IGF-I, which was only measured at baseline. The catheter was flushed with NaCl solution after each sampling. 

### 2.6. Laboratory Analyses

Fasting blood samples were drawn prior to the OGTTs before and after treatment for the analysis of a complete blood count, electrolyte status including creatinine, alanine amino transferase (ALAT), *γ*-glutamyl transpeptidase (*γ*-GT), cholesterol levels including high-density lipoprotein (HDL), low-density lipoprotein (LDL), triglycerides (TG), and HbA_1C_. HbA_1c_ was analyzed using the separation of HbA1c by HPLC, followed by analysis with Variant II Turbo Clinical Data Management software (BioRad Laboratories Inc., Hercules, CA, USA). A morning urine sample was collected for the analysis of microalbuminuria. All of the above were analyzed using standard, accredited methods at the Central Chemistry Laboratory at Karolinska University Hospital.

*Blood glucose* was analyzed from whole blood within 30 minutes from sampling directly in the research facility using YSI 2300 Stat Plus apparatus (Yellow Springs, OH, USA). 

*Serum cortisol* was analyzed at the Central Chemistry Laboratory at Karolinska University Hospital with chemoluminescence technique, using Roche Modular apparatus (Roche Diagnostics Scandinavia, Bromma, Sweden). The total coefficient of variation (CV) was 2.5% at 544 nmol/L and 2.1% at 855 nmol/L. 

Serum samples for analyses of insulin, C-peptide, IGF-I, and IGFBP1 were centrifuged and stored at −80°C until the completion of the study. This allowed all of the serum samples to be analyzed in the same batch. 

*Serum insulin* was analyzed with an in-house radioimmunoassay (RIA) method (using Pharmacia insulin RIA 100, Pharmacia Diagnostics, Uppsala, Sweden) [20]. The interassay CV was <5.8% and the intra-assay CV <5.4%.

*Serum C-peptide *was analyzed using human C-peptide RIA kit HCP-20 K (Millipore, Billerica, MA, USA). The detection level was 0.1 *μ*g/L and the performance level was ED_50_ at 1.1 ± 0.1 *μ*g/L, ED_80_ at 0.3 ± 0.1 *μ*g/L.

*Total serum IGF-I* was determined by an in-house RIA after the separation of IGFs from IGFBPs by acid ethanol extraction and cryoprecipitation [21]. The detection level of the RIA was 3.0 mg/L. Cross-reactivity with IGFBP-2 and IGFBP-3 was less than 0.5 and 0.05%, respectively. To minimize the interference of remaining IGFBPs, des (1–3) IGF-I was used as radioligand [21]. Serum levels of IGF-I decrease with age and are thus expressed as standard deviation (SD) score = [(10log⁡⁡IGF-I_observed_ + 0.00693∗age) − 2.581]/0.120 [22]. The intra- and interassay CVs were 4% and 11%, respectively. 

*Serum IGFBP1 *was also analyzed with an in-house RIA [23]. The sensitivity was 3 *μ*g/L and the intra- and interassay CVs were 3% and 10%, respectively. 

### 2.7. Calculations

Delta area under the curve (D-AUC) was used to compare AUC values during OGTT from different weeks of treatment, correcting for the baseline value. Homeostatic model of insulin resistance (HOMA-IR) was calculated as (serum insulin ∗ blood glucose)/22.5, using fasting values of glucose and insulin. 

### 2.8. Statistical Methods

Statistical analyses were carried out using the STATISTICA software, version 10 (StatSoft, Tulsa, OH, USA). *P* values <0.05 were considered statistically significant. Normality of variables was tested using the Kolmogorov-Smirnov and Lilliefors tests. Difference between variables that were normally distributed were analyzed using Student's *t*-test, whereas variables that were not normally distributed were analyzed using the Wilcoxon and Mann-Whitney tests. The baseline period of microdialysis was analyzed by repeated measures ANOVA of the measurements from −30 to +30 minutes.

## 3. Results

### 3.1. Clinical Characteristics before and after 12 Weeks of Treatment (Table 1)

Before treatment, subjects with T2D and IGT were similar in regards to BMI, waist circumference, and lipids. T2D patients were younger and had lower diastolic blood pressure but higher HbA_1c_. Their medications are summarized in Table 1. Treatment with pioglitazone did not significantly change HbA_1c_, weight, BMI, or WHR in either group. Total serum cholesterol, HDL, and LDL did not differ between the groups before or during treatment. Lipid levels did not differ between the groups before or during treatment, although fasting serum triglycerides decreased in the T2D group (*P* = 0.013). Serum *γ*-GT decreased in the T2D group (*P* = 0.021), while it was unchanged in the IGT group.

### 3.2. Effects on Glucose and Lipid Metabolism (Table 2)

Fasting glucose, C-peptide, and HOMA-IR decreased in both groups. However, fasting serum insulin decreased only in T2D. D-AUCs for blood glucose, insulin, and C-peptide during OGTT were unchanged, except for D-AUC for insulin in the IGT group which decreased (data not shown). 

### 3.3. Effects on the GH-IGF-I Axis

**Figure 1:**
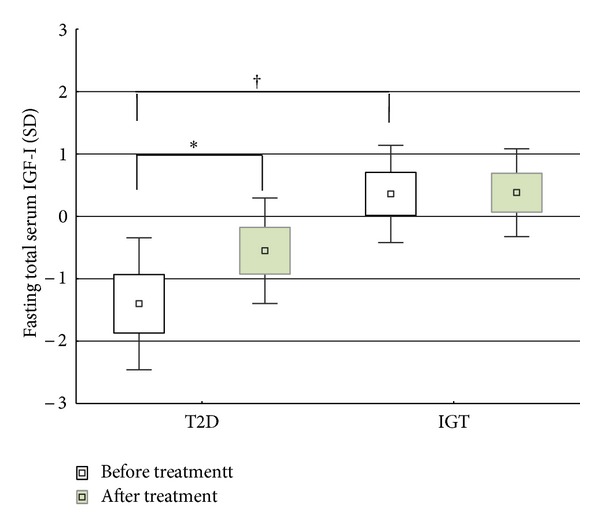
Fasting serum IGF-I before and during pioglitazone treatment in type 2 diabetes (T2D) and impaired glucose tolerance (IGT). ^†^Mann-Whitney *U*-test, *P* = 0.006. *Wilcoxon matched pairs test, *P* = 0.017.

Before treatment, total serum IGF-I was lower in the T2D group compared to the IGT group (*P* = 0.006; Figure 1). It increased after 12 weeks in the former (*P* = 0.017) to a level nearer the population mean, whereas it was unchanged in the latter. There was no change in D-AUC in either group. Serum IGFBP1 decreased during the OGTTs in both groups (*P* < 0.001 in both), but there was no change in the levels before and during treatment in either group. No correlations were found between any of the baseline parameters or their D-AUC. 

### 3.4. Adipose Tissue Glycolysis and Lipolysis Measured with Microdialysis during OGTT (Table 3)

Fasting interstitial glucose and pyruvate were higher in the T2D group before treatment (*P* = 0.005 and 0.010, resp.) but not afterwards. Glucose, pyruvate, and lactate were unchanged in both groups, while fasting glycerol decreased in the T2D group (*P* = 0.038). D-AUCs were unaffected save for an increase in interstitial lactate in the IGT group (*P* = 0.043). D-AUC interstitial glucose was higher in the T2D group both before and during treatment compared with the IGT group (*P* = 0.008 and 0.011, resp.), while no differences were noted in D-AUC of the other interstitial measurements.

### 3.5. Discrepant Effects on the HPA Axis in the T2D and IGT Group

**Figure 2:**
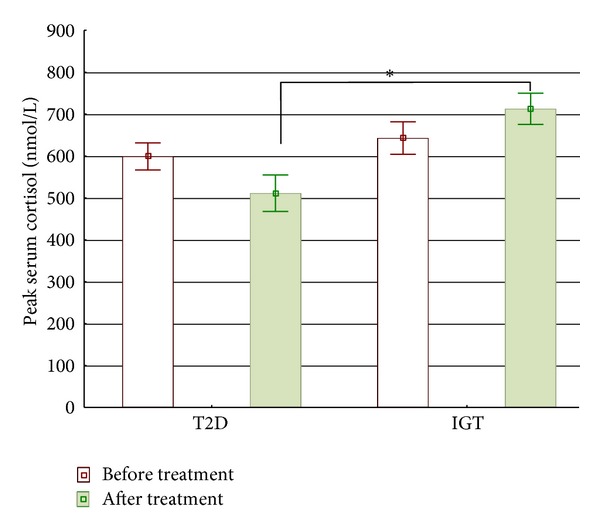
Peak serum cortisol (after 1 *μ*g ACTH injection) before and during pioglitazone treatment in type 2 diabetes (T2D) and impaired glucose tolerance (IGT). *Mann-Whitney *U*-test, *P* = 0.007.

Before treatment, there was no significant difference in fasting serum cortisol between the two groups. It decreased in the T2D group during treatment (*P* = 0.041), whereas it increased in the IGT group (*P* = 0.044), resulting in a difference between the groups at week 12 (*P* = 0.007; Table 2). Before treatment, there was no significant difference in peak cortisol between the groups, whereas during treatment it was lower in T2D (*P* = 0.007; Table 2 and Figure 2). There were no significant changes in glucose, insulin, C-peptide, or IGFBP1 levels after ACTH injection before and during pioglitazone treatment in either group (data not shown).

### 3.6. Effect of Glucose-Lowering Treatment

To investigate if treatment influenced the results of the study, the metformin-treated patients (*n* = 6) were compared to the rest of the diabetic patients (*n* = 4) despite groups being very small. HOMA still decreased in both groups, from 9.5 ± 2.0 to 5.3 ± 0.8 in the metformin group, *P* = 0.028 and from 6.3 ± 1.5 to 4.0 ± 0.7 in the nonmetformin group, *P* = 0.068. 

IGF-I increased in both the metformin-treated patients (from −1.8 ± 1.7 to −0.9 ± 1.3 SD, *P* = 0.068), and in the nonmetformin group (from −0.8 ± 1.0 to 0.0 ± 0.8 SD, *P* = 0.144). There was no trend toward a change in IGFBP1 in either subgroup.

The mean level of fasting cortisol decreased within each subgroup, from 383 ± 37 to 300 ± 41 nmol/L in the metformin group, *P* = 0.016 and from 426 ± 53 to 330 ± 13 nmol/L in the nonmetformin group, *P* = 0.144.

Peak cortisol decreased significantly in the metformin group (613 ± 43 to 449 ± 27 nmol/L, *P* = 0.028) but did not change in the nonmetformin group (578 ± 55 to 605 ± 87 nmol/L, *P* = 0.715). Baseline glycerol still decreased significantly in the metformin group (*P* = 0.036 ANOVA) but not in the nonmetformin group (*P* = 0.570 ANOVA).

## 4. Discussion

This study of patients with T2D and IGT is the first to use pioglitazone as a tool to study the interactions between insulin sensitivity and the IGF-I and HPA axes. Alongside improved insulin sensitivity in both groups, serum IGF-I increased in the T2D group only, while fasting serum cortisol decreased in T2D subjects and unexpectedly increased in IGT subjects, who displayed higher reactivity to low-dose ACTH compared to T2D subjects during treatment.

Fasting levels of glucose and C-peptide decreased as expected [24] in all patients during treatment, but fasting insulin only in the T2D group. However, HOMA-IR improved in both groups indicating improved hepatic insulin sensitivity [25]. A major factor separating the pathology of IGT and T2D is that the former displays high insulin levels to compensate insulin resistance, whereas at least in advanced T2D *β*-cell function and hence insulin levels are reduced while blood glucose and triglycerides are increased. Since the mean duration of known diabetes in the T2D patients was 4 years and none were treated with exogenous insulin, most likely *β*-cell function was impaired but not abolished. They also had good metabolic control from the start, which may explain the lack of significant decrease in HbA_1c_ during treatment. The T2D group responded to treatment with decreased triglyceride levels, whereas the IGT group did not; this may be an underlying cause of the discrepant responses to pioglitazone as discussed below. 

IGF-I increased during pioglitazone treatment in T2D but not IGT. IGF-I contributes to approximately 10% of total insulin sensitivity [26], so the increase may explain a small portion of the improved insulin sensitivity in the T2D group. However, it is more likely that the increase in IGF-I is a result of the improved adipose tissue insulin sensitivity as reflected by the reduced serum triglycerides and interstitial glycerol (reduced lipolysis) despite reduced levels of insulin, the main inhibitor of lipolysis. This would result in lower FFAs, a known effect of TZD treatment [15]. Lower FFAs would allow the increased GH levels and secondarily increased IGF-I [5]. IGF-I increased toward but not above the age-adjusted population mean, indicating a normalization of a deranged metabolic milieu. IGFBP1 levels were in the lower normal range before treatment and remained low during treatment despite decreased fasting serum insulin and C-peptide in the T2D group. This indicates an increase in free serum IGF-I and improved hepatic insulin sensitivity as insulin's inhibiting effect on the liver is the main factor regulating IGFBP1 levels [3]. 

GH is a powerful metabolic hormone. We did not measure GH levels, but would not expect them to be elevated as our patients were overweight or obese. The decreased lipolysis noted in the T2D group may have been due to decreased cortisol, or to increased IGF-I causing feedback inhibition of GH, which is lipolytic [5]. Along with reducing FFAs, TZDs redistribute fatty acids from the liver to the less metabolically active sc adipose tissue, reducing hepatosteatosis [15]. In the T2D group, decreased serum triglycerides and *γ*-GT are in accordance with this and may explain the improved hepatic insulin sensitivity, alongside the above effects on the IGF system. 

Triglycerides, interstitial glycerol, and insulin were unaffected in the IGT group, indicating that while hepatic insulin sensitivity improved, adipose tissue sensitivity was unaffected. This group entered the study with IGF-I levels nearer the population mean, which were not affected by treatment. Hence, the improvement of lipid metabolism that occurred in the T2D group was absent in the IGT group, as was the concomitant effect on the GH-IGF-I axis.

Interstitial measurements also showed higher glucose and pyruvate before treatment in the T2D group, indicating mitochondrial dysfunction [27]; however, treatment had no effect on interstitial measurements reflecting glycolysis. This may have been due to short treatment duration. There was an isolated increase in D-AUC of interstitial lactate during the OGTT in the IGT group. Lactate production is increased in obesity, also possibly due to mitochondrial dysfunction [28]. The reduction in interstitial lactate during OGTT may indicate an increased utilization of glycolysis products by the mitochondria, previously shown during pioglitazone treatment in T2D patients [29].

This study was designed to evaluate whether the inhibition of the HPA axis that pioglitazone treatment caused in rodents also occurred in humans. The results in the T2D group of this study support that such may be the case. The subjects in the T2D and IGT groups had similar fasting cortisol levels before treatment, which subsequently decreased and increased, respectively, during treatment. Changes in peak cortisol levels were not significant within either group, possibly due to a small number of subjects, but a trend toward a decrease in the T2D group and an increase in the IGT group resulted in a significant difference in peak cortisol between the groups during treatment. We have no doubts that the ACTH levels obtained were sufficient for stimulating cortisol release in the IGT group and consider the lack of response in the DM group as a pathologic reaction to a sufficient stimulation. 

Basal cortisol is influenced both by the activity of the HPA axis, peripheral synthesis of cortisol from inactive cortisone via the enzyme 11*β*-hydroxysteroid dehydrogenase type 1 (11*β*HSD1) in various organs, and peripheral metabolism and excretion. As only the HPA axis was evaluated in this study, no conclusion can be drawn on the cause of the change in basal cortisol. GH inhibits 11*β*HSD1 in the liver through an IGF-I mediated effect [30]; thus, its activity in the T2D group may have been reduced as IGF-I increased, resulting in a reduced basal cortisol. However, the increase in basal cortisol in the IGT group was unrelated to any change in IGF-I; further study will be required to elucidate the cause of this finding.

It has previously been shown that the 1 *μ*g ACTH test is more sensitive than the 250 *μ*g test in testing the sensitivity of the adrenal cortex to ACTH [31]. In studies of the peak cortisol response to 1 *μ*g Synacthen in healthy subjects, means between 466 and 600 nmol/L have been reported [32–34] with peak levels of >400 nmol/L considered as within normal range. Based on these results, none of the subjects displayed signs of adrenal insufficiency; peak cortisol levels ranged from 460 to 764 nmol/L. There was a trend toward decreased adrenal cortex sensitivity in the T2D group and increased sensitivity in the IGT group, although the data was not significant. These changes were most likely not related to altered IGF-I levels, as previous studies have found no effect on ACTH or cortisol responses to stimulation tests in subjects treated with recombinant IGF-I [35]. 

There are limitations to this study. Being a pilot study aimed at generating new hypotheses for future studies, only small groups were studies and no women were included. However, the main findings of the study were consistent when analyzing the 6 patients treated with metformin, that is, decreased HOMA-IR, increased IGF-I, decreased peak cortisol, and baseline glycerol after pioglitazone treatment. HOMA-IR, which was used to characterize insulin sensitivity, is best suited for the study of larger populations. However, as no patient was treated with any medication that directly affected fasting glucose and insulin HOMA-IR was used to estimate the intraindividual change in insulin sensitivity due to pioglitazone. Measurement of serum FFAs would have further increased the strength of our conclusion regarding lipolysis, but this was omitted mainly as previous studies consistently have shown FFA levels to decrease with pioglitazone treatment [15]. 

## 5. Conclusion

In summary, insulin sensitivity improved in both groups but adipose tissue sensitivity only in the T2D group. This was accompanied by increased IGF-I and decreased fasting serum cortisol in the T2D group alone. Surprisingly, there was instead an increase in fasting and stimulated serum cortisol levels in the IGT group. The differences between the T2D and IGT groups were most evident for the metformin-treated patients. While the reaction of the GH–IGF-I axis may be attributed to improved lipid metabolism the discrepant reactions of cortisol seen in T2D and IGT will require further studies with larger groups including both genders and patients with poor metabolic control.

## Figures and Tables

**Table 1:** Subject characteristics and medications prior to pioglitazone treatment (mean ± SEM).

	T2D (*n* = 10)	IGT (*n* = 10)	*P*
Age (years)	54 ± 2	61 ± 2	0.031
Weight (kg)	97.7 ± 3.4	94.1 ± 3.0	0.436
BMI (kg/m^2^)	31.0 ± 0.7	29.5 ± 0.4	0.105
Waist circumference (cm)	111 ± 3	106 ± 2	0.315
Systolic blood pressure (mmHg)	136 ± 5	146 ± 4	0.138
Diastolic blood pressure (mmHg)	84 ± 2	89 ± 2	0.040
Triglycerides (mmol/L)	1.5 ± 0.2	1.1 ± 0.1	0.073
Low-density lipoprotein (mmol/L)	3.1 ± 0.3	3.3 ± 0.4	0.723
High-density lipoprotein (mmol/L)	1.1 ± 0.1	1.2 ± 0.2	0.624
Gamma-GT (*μ*kat/L)	0.8 ± 0.2	0.5 ± 0.1	0.233
HbA1c (mmol/mol)	57 ± 4 (*n* = 9)	41 ± 1	0.006
Duration of disease (years)	4 ± 1	n.a.	n.a.

Metformin (*n*)	6		
Repaglinide (*n*)	1		
Acarbose (*n*)	1		
Low-dose aspirin (*n*)	1	1	
Statin (*n*)	3	2	
ACE-inhibitor (*n*)	2	2	
Angiotensin receptor blocker (*n*)	1	2	
Thiazide diuretic (*n*)	1	1	
Beta-blocker (*n*)	1		

**Table 2:** Fasting values in 10 subjects with type 2 diabetes (T2D) and 10 subjects with impaired glucose tolerance (IGT) at baseline (week 0) and after 12 weeks of pioglitazone treatment (mean ± SEM).

	T2D				IGT				T2D versus IGT		
	Week 0	Week 12	Δ week	*P* week	Week 0	Week 12	Δ week	*P* week	P week 0	*P* week 12	*P* for Δ week
Blood glucose (mmol/L)	8.7 ± 0.8	6.9 ± 0.4	−1.7 ± 0.5	0.007	5.0 ± 0.2	4.7 ± 0.1	−0.3 ± 0.1	0.028	0.001	0.002	0.015
Serum insulin (mU/L)	20.5 ± 2.4	15.3 ± 1.4	−5.2 ± 1.6	0.011	26.5 ± 7.0	24.4 ± 7.5	−2.8 ± 6.6	0.263	0.912	0.780	0.447
HOMA-IR	8.2 ± 1.4	4.8 ± 0.6	−3.4 ± 1.0	0.005	5.9 ± 1.5	4.9 ± 1.4	−1.2 ± 0.5	0.038	0.123	0.604	0.072
Serum C-peptide (*μ*g/L)	1.6 ± 0.3	1.3 ± 0.3	−0.3 ± 0.1	0.008	2.8 ± 0.4	2.0 ± 0.3	−0.8 ± 0.2	0.010	0.105	0.190	0.105
Serum IGF-I (SD)	−1.4 ± 0.5	−0.5 ± 0.4	0.9 ± 0.2	0.017	0.4 ± 0.3	0.4 ± 0.3	0.0 ± 0.2	0.913	0.006	0.143	0.019
Serum IGFBP1 (*μ*g/L)	18.4 ± 4.1	20.8 ± 4.7	2.4 ± 2.9	0.760	18.4 ± 2.2	21.8 ± 4.0	3.4 ± 3.3	0.326	0.436	0.684	0.821
Serum cortisol, baseline (nmol/L)	400 ± 30	312 ± 25	−88 ± 37	0.047	402 ± 21	461 ± 35	58 ± 25	0.014	0.853	0.007	0.004
Serum cortisol, peak (nmol/L)	599 ± 32	511 ± 43	−88 ± 56	0.155	643 ± 39	713 ± 37	70 ± 40	0.114	0.395	0.007	0.035
Serum triglycerides (mmol/L)	1.5 ± 0.2	1.1 ± 0.1	−0.3 ± 0.2	0.021	1.1 ± 0.1	1.1 ± 0.2	0.0 ± 0.2	0.959	0.073	0.970	0.247

**Table 3:** Interstitial measurements in adipose tissue during OGTT in 10 subjects with type 2 diabetes (T2D) and 10 subjects with impaired glucose tolerance (IGT) at baseline (week 0) and after 12 weeks of pioglitazone treatment (mean ± SEM).

	T2D						IGT						T2D versus IGT			
	Baseline week 0	Baseline week 12	*P* ANOVA*	D-AUC week 0	D-AUC week 12	*P*	Baseline week 0	Baseline week 12	*P* ANOVA*	D-AUC week 0	D-AUC week 12	*P*	*P* baseline week 0	*P* baseline week 12	*P*D-AUC week 0	*P* D-AUC week 12
Glucose (mmol/L)	8.2±0.9	8.1 ± 0.6	0.269	655 ± 107	630 ± 100	0.600	4.6 ± 0.6	6.8 ± 0.6	0.210	405 ± 60	257 ± 81	0.161	0.005	0.169	0.008	0.011
Pyruvate (*μ*mol/L)	210.1 ± 10.7	145.7 ± 22.7	0.073	6368 ± 1337	4270 ± 607	0.785	127.9 ± 22.2	175.7 ± 34.0	0.384	6649 ± 1415	2338 ± 1222	0.100	0.010	0.965	0.962	0.175
Lactate (mmol/L)	2.1 ± 0.2	1.8 ± 0.2	0.454	101 ± 18	87 ± 9	0.773	2.0 ± 0.3	1.6 ± 0.1	0.157	64 ± 26	108 ± 18	0.043	0.791	0.842	0.236	0.333
Glycerol (*μ*mol/L)	296.3 ± 32.4	192.0 ± 21.8	0.038	−11451 ± 1527	−7885 ± 1138	0.167	264.3 ± 36.5	256.2 ± 34.3	0.846	−10130 ± 2807	−9998 ± 8304	0.917	0.910	0.198	0.903	0.860

*Repeated measures ANOVA over the baseline period, for example, from −30 to + 30 minutes.
